# Assessment of the feasibility of same-day discharge following minimally invasive hysterectomy in the elderly population

**DOI:** 10.1016/j.gore.2023.101227

**Published:** 2023-06-19

**Authors:** Paulina J Haight, Rachael N Piver, David A Barrington, Jae Baek, Stephen M Graves, Melissa Ardizzone, Jenifer A Akinduro, Audrey C Busho, Deborah Fadoju, Radhika Pandit, Raeshawn Stephens, Lauren M Strowder, Shreekari Tadepalli, Brianna VanNoy, Bhargavi Sriram, Eric M McLaughlin, Michelle DS Lightfoot, Laura M Chambers, Kristin L Bixel, David E Cohn, Casey M Cosgrove, David O'Malley, Ritu Salani, Floor J Backes, Christa I Nagel

**Affiliations:** a Division of Gynecologic Oncology, Department of Obstetrics and Gynecology, The Ohio State University Wexner Medical Center, James Hospital and Solove Research Institute, Columbus, OH, USA; b Department of Obstetrics and Gynecology, The Ohio State University Wexner Medical Center, Columbus, OH, USA; c The Ohio State University College of Medicine, Columbus, OH, USA; d Center for Biostatistics, The Ohio State University College of Medicine, Columbus, OH, USA

**Keywords:** Minimally invasive hysterectomy, Same-day discharge, Elderly, Frailty

## Abstract

**Objective:**

To determine the safety and feasibility of same-day discharge (SDD) following minimally invasive hysterectomy (MIH) for elderly patients and to evaluate associations between age, frailty, and postoperative outcomes.

**Methods:**

Retrospective review was conducted of patients aged ≥ 70 who underwent MIH within a single gynecologic oncology institution from 2018 to 2020. Demographics, *peri*-operative factors, postoperative complications, and 30-day readmission rates were collected. Frailty was determined by an 11-point modified frailty index ≥ 2. Outcomes were compared between SDD and observation groups using Fisher’s exact and Wilcoxon rank-sum tests.

**Results:**

Of 169 patients included in the analysis, 8.9% (n = 15) underwent SDD, and 91.1% (n = 154) were admitted for OBS following MIH. Demographics, *peri*-operative factors, and frailty rates (33% SDD vs 43.5% observation; p = 0.59) were similar between groups. 86.7% (n = 13) of SDD cases were completed before 12PM, and none were completed after 6PM. No SDD patients had early post-operative complications or hospital readmissions. Early postoperative complications were diagnosed in 9 (5.8%) patients admitted for OBS, and the 30-day hospital readmission rate for patients who underwent OBS was 8.4% (n = 13). While elderly patients who met objective frailty criteria (n = 72) did not have a higher likelihood of early post-operative complications (44.4% vs 55.6%; p = 0.909), they did have a higher likelihood of ED visit within 30 days of discharge (15.3 vs 3.1%; p = 0.009), and a trend was noted toward a higher rate of 30-day hospital readmission (12.5% vs 4.1%; p = 0.080).

**Conclusions:**

Elderly patients undergoing SDD following MIH did not have increased morbidity or mortality. Elderly patients who meet objective criteria for frailty, however, represent a more vulnerable population.

## Highlights

• Advanced age alone should not serve as contraindication to same-day discharge following minimally invasive hysterectomy.
• Elderly patients who undergo same-day discharge are not at increased risk of 30-day hospital readmission.
• Elderly, frail patients encompass a higher-risk group where prehab and heightened *peri*-operative monitoring are warranted.

## 1 Introduction

Hysterectomy is one of the most common surgical procedures performed, with over 600,000 cases in the United States annually (Aloisi et al., 2020). Forty percent of hysterectomies are now completed via a minimally invasive approach, which has increased significantly over the past 20 years (ACOG Committee, 2018, Bixel et al., 2022). Most women who undergo surgery for endometrial cancer undergo a minimally invasive hysterectomy (MIH), and the oncologic outcomes of this approach are comparable to laparotomy (Bishop et al., 2018, Brancazio et al., 2021, Hospital, 2022). Additionally, compared to open surgery, MIH is associated with numerous benefits, including lower rates of *peri*-operative complications, faster recovery time, and decreased length of hospital stay (Di Donato et al., 2021, Farhat et al., 2012, Fountain and Havrilesky, 2017, Gallotta et al., 2018).

Contemporary practice trends have incorporated same-day discharge (SDD) at the time of MIH (Praiss et al., 2019). Existing literature for benign and oncologic MIH suggests that SDD is safe and not associated with an increased risk of adverse outcomes compared to patients observed overnight (OBS) (Di Donato et al., 2021, Farhat et al., 2012, Gallotta et al., 2018, Harris et al., 2009 Apr). SDD is also cost-effective relative to OBS and is associated with improved patient satisfaction scores (Farhat et al., 2012, Gallotta et al., 2018, Harris et al., 2019, Janda et al., 2017). Many factors play a role in the decision for SDD, including patient characteristics, *peri*-operative considerations, and surgeon and patient preference. Factors that have been shown to increase the likelihood of OBS following MIH include completion of surgery after 1PM, intra-operative complications, significant medical comorbidities, lack of social support, and greater distance between home and hospital (Di Donato et al., 2021, Janda et al., 2017, Jennings et al., 2015, Rivard et al., 2015).

Many sources caution that advanced age (≥70-80 years) should be a relative contraindication to SDD (Janda et al., 2017, Jennings et al., 2015, Krause et al., 2016, Korsholm et al., 2017). With the increasing rate of endometrial cancer, the age of the gynecologic oncology patient population undergoing MIH is expected to continue to rise. An additional consideration is that while historically, elderly patients were considered to have decreased tolerance for surgical management, recent data supports that other factors aside from chronological age, including frailty assessments, should be considered in treatment planning. We sought to assess the safety of SDD in an elderly (age ≥ 70) gynecologic oncology population by comparing rates of early postoperative complications and unscheduled presentations to care, including outpatient problem visits, emergency room (ED) visits, and hospital readmissions within 30 days of discharge for patients who underwent SDD versus OBS. Additionally, we evaluated the association between frailty and post-operative outcomes, including early post-operative complications, ED visits, and 30-day hospital readmission rates, in a cohort of elderly patients undergoing MIH.

## 2 Methods

The study was approved by the Institutional Review Board and the Comprehensive Cancer Center Clinical Scientific Review Committee at The Ohio State University (Institutional Review Board No. 2018C0142). Charts from patients who underwent MIH within the gynecologic oncology division from January 2018 – August 2020 were retrospectively reviewed. The decision for SDD vs OBS was primarily based on surgeon discretion following *peri*-operative counseling and shared decision-making with the patient without randomization. Only patients aged 70 or older were included in the analysis. Patients were excluded from the study for abortion of hysterectomy or conversion to laparotomy, in addition to the unavailability of records. Demographics, *peri*-operative factors, postoperative complications, unscheduled presentations, and readmissions within 30 days of discharge following MIH were collected and managed using REDCap electronic data capture tools hosted by The Ohio State University (Lin et al., 2016, Nahas et al., 2016). A portion of this cohort was previously published (Haight et al., 2023).

The Charlson Comorbidity Index (CCI) was calculated for each patient as a mortality prediction tool. Modified Frailty Index (mFI) was calculated for each patient using the 11-factor schema that was created by comparing the original Canadian Study of Health and Aging Frailty Index (CSHA-FI) with variables measured by the National Surgical Quality Improvement Program (NSQIP) (Staff, 2022). Frail patients were classified as those with mFI scores of 2 or higher, consistent with prior studies (Nguyen et al., 2021, Rockwood et al., 2011). Patient demographics, surgical details, *peri*-operative complications, and follow-up outcomes were compared between patients who underwent SDD and those admitted for OBS. The primary outcome was unscheduled presentation within 30 days of discharge for patients undergoing SDD versus OBS. Secondary outcomes assessed were early postoperative complications, or those diagnosed within 48 h of surgery, and routine monitoring for patients undergoing OBS. Additionally, all post-operative outcomes and unscheduled presentations for evaluation within 30 days of discharge were compared between non-frail (mFI 0–1) versus frail (mFI ≥ 2) patients. Categorical variables and outcomes were compared between discharge status using Fisher’s exact tests. Continuous variables were assessed for normality using Kolmogorov-Smirnoff tests. Due to the non-normal distributions, continuous data are presented as median [first-third quartiles] and compared using Wilcoxon rank-sum tests. All statistical analysis was performed using SAS version 9.4 (SAS Institute, Inc., Cary, NC).

## 3 Results

Of the 1,160 patients underwent MIH within the gynecologic oncology division of our institution from 2018 to 2020. One hundred and sixty-nine were age ≥ 70 and were included in the analysis. No patients were enrolled in a prehabilitation program preoperatively. Fifteen (8.9%) patients underwent SDD, and 154 (91.1%) patients underwent OBS following MIH. There were no significant differences in patient demographics or medical co-morbidities between those patients who underwent SDD versus OBS (Table 1 and Table 2). A trend was noticed toward younger age, current employment, and cohabitation with another adult for those patients who underwent SDD compared to OBS, however, this was not statistically significant. Seventy-two (42.6%) patients met objective criteria for frailty based on mFI ≥ 2. Frailty did not significantly differ between the SDD versus OBS groups (33.3% vs 43.5%, respectively; p = 0.59).

**Table 1:** Patient demographics.

	**SDD(n = 15)**	**OBS(n = 154)**	**P-value**
Race*			0.53
White	14 (93.3)	146 (95.4)	
Non-white	1 (6.7)	7 (4.6)	
Ethnicity			0.36
Hispanic	1 (7.1)	4 (2.6)	
Non-Hispanic	13 (92.9)	150 (97.4)	
Less than 60 miles from OSU*	6 (40.0)	92 (60.1)	0.17
Insurance*			1.0
Private	0 (0)	1 (0.7)	
Medicare	15 (100)	146 (98.0)	
Medicaid	0 (0)	1 (0.7)	
Self-pay	0 (0)	1 (0.7)	
Employed*			0.054
Yes	3 (20.0)	9 (6.0)	
No	3 (20.0)	17 (11.3)	
Retired	9 (60.0)	124 (82.7)	
Living situation			0.06
Alone	1 (7.7)	42 (35.9)	
With 1 or more others	12 (92.3)	75 (64.1)	
Tobacco use			1.0
Never	11 (73.3)	105 (68.2)	
Current	0 (0)	9 (5.8)	
Former	4 (26.7)	40 (26.0)	
Alcohol use	3 (20.0)	46 (29.9)	0.56
History of chemotherapy*	1 (6.7)	6 (3.9)	0.48
History of radiation*	0 (0)	2 (1.3)	1.0
Age at surgery (years)	73 [71–76]	75 [72–80]	0.11
BMI	34 [26–40]	33 [28–40]	0.83
CCI	5 [3–8]	6 [5–8]	0.22
mFI ≥ 2	5 (33.3)	67 (43.5)	0.59
# prior abdominal surgeries*	1 [0–2]	1 [0–2]	0.38

Abbreviations: SDD, same day discharge; OBS, overnight observation; BMI, body mass index (kg/m^2^); CCI, Charlson Comorbidity Index; mFI, Modified Frailty Index.

*Indicates some patients were missing data for given variable.

Results are reported as frequency (column percent) with chi-square or Fisher’s exact p-value, or median [first-third quartiles] and Wilcoxon rank-sum test.

**Table 2:** Patient medical co-morbidities.

	**SDD(n = 15)**	**OBS(n = 154)**	**P-value**
None	3 (20.0)	16 (10.4)	0.38
Hypertension	10 (66.7)	118 (76.6)	0.36
Myocardial infarction	0 (0)	6 (3.9)	1
PCI	2 (13.3)	14 (9.1)	0.64
CHF	0 (0)	8 (5.2)	1
PVD	1 (7.1)	9 (5.8)	1
Stroke	2 (13.3)	17 (11.0)	0.68
Dementia	0 (0)	4 (2.6)	1
COPD	2 (13.3)	8 (5.2)	0.22
OSA or use of CPAP	3 (20.0)	24 (15.6)	0.71
Diabetes	2 (13.3)	41 (26.6)	0.36
Moderate/severe CKD	2 (13.3)	24 (15.6)	1
PUD	1 (6.7)	5 (3.3)	0.43
Liver disease	0 (0)	1 (0.7)	1
Leukemia/lymphoma	0 (0)	2 (1.3)	1
Localized tumor	7 (46.7)	89 (57.8)	0.43
Metastatic tumor	3 (20.0)	27 (17.5)	0.73
ECOG > 2	0 (0)	3 (2.0)	1
Other significant comorbidities	3 (20.0)	36 (23.4)	1

Abbreviations: SDD, same day discharge; OBS, overnight observation; PCI, percutaneous coronary intervention; CHF, congestive heart failure; PVD, peripheral vascular disease; COPD, chronic obstructive pulmonary disease; OSA, obstructive sleep apnea; CPAP, continuous positive airway pressure; CK, chronic kidney disease; PUD, peptic ulcer disease.

Results are reported as frequency (column percent) with chi-square or Fisher’s exact p-value.

The most common indication for MIH was endometrial cancer. Other indications included complex atypical hyperplasia, pelvic mass, and cancer risk reduction; these did not differ significantly between the two groups. All patients underwent concurrent salpingectomy. Most patients underwent lymph node assessment (n = 128, 75.7%). Rate of SDD did not differ based upon type of lymph node assessment (7.5% none, 13.2% sentinel, 3.2% full pelvic, 4.6% pelvic and *para*-aortic; p = 0.3143). Additional surgical staging or debulking procedures were performed in 36 (21.3%) cases, most commonly omentectomy and peritoneal biopsies (n = 14); this did not differ significantly between the two groups. All patients who required mini-laparotomy for specimen removal (n = 17, 10.1%) and who had an intra-operative complication (n = 4, 2.4%) were admitted for OBS. Intra-operative complications included ureter injury (n = 1), bladder injury (n = 1), and estimated blood loss > 500 ml (n = 2).

Same day discharge rates differed based on time of surgical completion. All patients with a surgery completion time after 6PM were admitted for OBS. Of the patients that underwent SDD, 13 (86.7%) cases were completed prior to 12PM, and 2 (13.3%) cases were completed between 12 and 6PM (p = 0.004). SDD rates per surgeon within our division ranged from 3.4 to 16.0%, and the rate of SDD did not significantly differ based on surgeon (p = 0.6526).

Of the patients admitted for OBS, 5.8% (n = 9) were diagnosed with an early post-operative complication (Table 3). These included infection, ICU admission, significant AKI, ileus, hyperglycemia requiring insulin drip, acute blood loss anemia requiring transfusion, significant electrolyte abnormalities, cardiopulmonary, and neurovascular event. All patients with an early post-operative complication had a pre-operative diagnosis of malignancy. Four (44.4%) patients were frail, and rates of early post-operative complications did not significantly differ according to frailty status (Table 4). The median day of discharge for patients who had an early post-operative complication was post-operative day 2 (range 1–11).

**Table 3:** Early post- operative complications, perioperative factors and outcomes among overnight observation patients.

**Case #**	**Age**	**BMI**	**HTN**	**DM**	**Cancer dx**	**mFI**	**Intra-operative complication**	**Telemetry monitoring**	**Pulse-ox monitoring**	**Early post-op complication**	**Discharge POD#**	**Hospital readmission?**
1	70	47	yes	yes	yes	2	no	yes	yes	Infectious, ICU admission, AKI, ileus, hyperglycemia requiring insulin gtt	11	yes
2	70	27	yes	no	yes	2	no	no	no	Acute blood loss anemia, significant electrolyte abnormalities	4	no
3	71	44	yes	no	yes	1	no	yes	yes	Acute blood loss anemia	2	no
4	71	28	yes	yes	yes	2	no	no	no	Significant electrolyte abnormalities	1	no
5	72	41	yes	no	yes	1	no	no	no	Neurovascular event	1	no
6	75	29	no	no	yes	1	no	yes	yes	Cardiopulmonary	1	no
7	83	32	yes	no	yes	1	no	yes	yes	Acute blood loss anemia, significant electrolyte abnormalities	2	no
8	85	29	yes	no	yes	1	no	yes	no	Cardiopulmonary	2	no
9	88	40	yes	yes	yes	2	no	yes	yes	Cardiopulmonary, infectious, ileus	7	yes

Abbreviations: BMI, body mass index (kg/m^2^); HTN, hypertension; DM, diabetes mellitus; dx, diagnosis; mFI, modified frailty index; POD#, post-operative day number.

**Table 4:** Post-operative complications and unscheduled presentations for evaluation according to modified frailty index.

**mFI**	**Post-op complication (n = 9)**	**P-value**	**ED visit within 30 days of discharge**	**P-value**	**Hospital readmission within 30 days of discharge**	**P-value**
0–1	5 (55.6)	0.909	3 (3.1)	**0.009**	4 (4.1)	0.080
≥ 2	4 (44.4)		11 (15.3)		9 (12.5)	

Abbreviations: mFI, modified frailty index; ED, emergency department.

Approximately one-third of the patients who underwent OBS underwent routine *peri*-operative monitoring with telemetry and/or pulse oximetry (n = 49 [32.4%] and n = 50 [32.9%], respectively) during admission. There was a trend toward heightened monitoring for frail patients who underwent OBS with telemetry (24% not frail, vs 38.6% frail; p = 0.06) and pulse-oximetry (21.6% not frail, vs 42.9% frail; p = 0.004). A rhythm abnormality was detected on telemetry in 6 (12.2%) patients, most of whom (n = 4, 66.7%) were frail. Five of the six patients with telemetry abnormalities required acute intervention with either IV antiarrhythmic medication and/or cardiology consultation; one of these patients was started on a beta-blocker that was continued at discharge. The remaining patient was asymptomatic, and the transient abnormality required no intervention. Oxygen desaturation <85% was detected on pulse-oximetry for 2 (4.0%) patients, both of whom were frail and managed with supplemental oxygen. One patient required oxygen supplementation at time of discharge.

None of the patients who underwent SDD presented for an outpatient problem visit, ED visit, or were readmitted to the hospital within 30 days of discharge. Of the 154 patients that were initially admitted to the hospital for OBS, 6 (4.2%) patients presented for an outpatient problem visit, 14 (9.2%) patients presented to the ED, and 13 (8.4%) were readmitted to the hospital within 30 days of discharge (Table 5). The median date of readmission was post-operative day 11 (range 2–30), and no patient was readmitted prior to post-operative day 2. The average age of those patients readmitted was 70, and the average CCI was 6.2. Two (15.4%) patients who were readmitted had a complicated early post-operative course prior to discharge, and most patients (n = 9, 69.2%) who were readmitted met objective criteria for frailty.

**Table 5:** Unscheduled presentation for evaluation within 30 days of discharge.

	**SDD(n = 15)**	**OBS(n = 154)**	**P-value**
No. of unscheduled outpatient visits within 30 days of discharge			1.0
0	15 (1 0 0)	139 (95.9)	
1	0 (0)	5 (3.5)	
2	0 (0)	1 (0.7)	
3	0 (0)	0 (0)	
ED visit within 30 days of discharge*	0 (0)	14 (9.2)	0.62
Hospital readmission within 30 days of discharge	0 (0)	13 (8.4)	0.61
Pain	0 (0)	3 (2.0)	1.0
SBO	0 (0)	2 (1.3)	1.0
Abdominal/pelvic abscess	0 (0)	4 (2.6)	1.0
Surgical take-back	0 (0)	4 (2.6)	1.0
AKI	0 (0)	3 (2.0)	1.0
UTI	0 (0)	1 (0.7)	1.0
Pneumonia	0 (0)	2 (1.3)	1.0
Cardiopulmonary	0 (0)	2 (1.3)	1.0
Neurovascular	0 (0)	1 (0.7)	1.0

Abbreviations: SDD, same day discharge; OBS, overnight observation; SBO: small bowel obstruction; AKI: acute kidney injury; UTI: urinary tract infection.

*Indicates some patients were missing data for given variable, or that data was difficult to interpret from the medical record.

## 4 Discussion

In this retrospective series of a large gynecologic oncology practice at a tertiary care center, elderly patients who underwent SDD following MIH did not have increased risk of early post-operative complications, outpatient problem visits, ED visits or hospital readmissions within 30 days of discharge compared to OBS. No patients who underwent SDD were readmitted to the hospital, whereas the readmission rate for OBS patients was 8.4%, with a median date of readmission being post operative day 11. There were trends toward worse post-operative outcomes including abnormal routine *peri*-operative monitoring and more frequent unscheduled presentations for evaluation in elderly frail patients compared to non-frail patients. Further, elderly patients who were frail had increased incidence of ED visits postoperatively. Patients who underwent OBS experienced a relatively low rate of early post-operative complication, and routine telemetry and pulse-oximetry monitoring in the *peri*-operative period resulted in an overall low incidence of clinical intervention, with most patients who required intervention meeting objective frailty criteria.

Existing literature supports the safety of MIH for elderly women, citing no increased risk of *peri*-operative morbidity or unscheduled presentation after discharge when compared to younger patients undergoing the same procedure (Nelson et al., 2019, Nensi et al., 2018 Jan, Melamed et al., 2016). Many sources also advocate for feasibility of SDD following MIH in the general population (Di Donato et al., 2021, Farhat et al., 2012, Gallotta et al., 2018, Harris et al., 2009 Apr). However, certain patient factors, including poorly controlled medical co-morbidities, morbid obesity, obstructive sleep apnea, surgical completion after noon, and lack of assistance at home are frequently cited as contraindications to SDD (Di Donato et al., 2021, Fountain and Havrilesky, 2017, Janda et al., 2017, Jennings et al., 2015). Older age (≥70-80) is also often listed as either a relative or absolute contraindication to SDD following MIH, though few studies to our knowledge have specifically looked at age as an independent predictive factor of SDD outcomes (Fountain and Havrilesky, 2017, Gallotta et al., 2018, Harris et al., 2009 Apr, Harris et al., 2019, Janda et al., 2017, Korsholm et al., 2017, Nensi et al., 2018 Jan). Our study suggests that age ≥ 70 should not serve alone as an absolute contraindication in an otherwise well-suited candidate for SDD following MIH, which is important to consider given the increasing rate of endometrial cancer and thus the number of patients eligible for MIH (Penner et al., 2015). No patients who underwent SDD in our cohort had an early post-operative complication that would have been detected by OBS, or hospital re-admission within 30 days of discharge.

The hospital readmission rate in our study was 8.4%, which is consistent with prior literature citing readmission rates of 0–17% following MIH (Farhat et al., 2012, Harris et al., 2009 Apr, Sanabria et al., 2019). Of note, most studies have found similar readmission rates between patients who underwent SDD versus those admitted for OBS, whereas none of our SDD patients were readmitted to the hospital within 30 days of discharge. Most patients who were readmitted (n = 9, 69.2%) were frail, and few readmitted patients (n = 2, 15.4%) had an early post-operative complication detected during OBS. This is different than The Laparoscopic Hysterectomy Readmission Score published by Jennings et al, where *peri*-operative complication weighs most heavily in the algorithm for risk-stratification for all patients who undergo MIH (Jennings et al., 2015). In addition to a higher 30-day hospital readmission rate (12.5% vs 4.1%), frail patients in our cohort tended to have more frequent abnormalities on telemetry and pulse-oximetry requiring intervention (telemetry: 14.8% vs 8.7%; pulse-oximetry: 100% vs 0%) and ED visits within 30 days of discharge (15.3% vs 3.1%) than non-frail patients. For elderly patients, it is possible that frailty may need to be a stronger consideration for perioperative risk assessment. Prehabilitation may be particularly beneficial in this setting. A recent *meta*-analysis of 15 randomized control trials to be feasible and associated with significant reduction in overall morbidity (OR 0.63) and pulmonary morbidity (OR 0.4) in patients undergoing major abdominal surgery (Hughes et al., 2019). While prehabilitation for all patients undergoing major surgery is ideal, a more reasonable strategy may consist of triaging vulnerable populations into a prehab program. Utilization of pre-operative assessment tools such as an objective frailty calculator may help the surgeon to identify some of these higher-risk groups, and potentially mitigate morbidity by recommending prehab entry prior to surgery. Additional heightened awareness of these patients in the post-operative setting, including earlier scheduled post-operative visits, may be beneficial.

Ultimately, physician counseling, patient health optimization, and prioritized morning scheduling for patients who are candidates for SDD may minimize healthcare costs associated with admission without compromising patient outcomes (Harris et al., 2019, Uppal et al., 2015). Prospective studies that assess qualitative data regarding surgeon and patient preferences may offer additional insight into the decision for SDD vs OBS in elderly patients following MIH. Subsequent educational and potentially social interventions could address barriers to SDD in otherwise appropriate candidates.

## 5 Strengths and limitations

There is a growing body of evidence that supports the safety of SDD following MIH. However, the vast majority of data is retrospective, and data on smaller, vulnerable groups such as the elderly is limited. In addition, there are very few studies that assess chronologic age and frailty as a sole predictor of outcomes of SDD after MIH. The strengths of our study include addition of information to a relatively understudied patient population, and the inclusion of objective measures of frailty and medical co-morbidity data. This provides further insight into risk factors for post-operative complications and unscheduled presentations after discharge for patients who undergo SDD versus OBS.

Limitations of our study include its retrospective nature, small sample size, and low rate of complications and readmissions. Further, given that decision for SDD was determined by the primary surgeon without randomization, the potential for selection bias favoring those at increased risk for medical and postoperative complications cannot be ignored. We suspect that this contributed to the improved functional status of the patients who underwent SDD. Additionally, given the retrospective design, we were unable to assess whether physician counseling and patient preference for disposition planning was considered. However, despite these limitations, our study provides proof-of-concept data that SDD following MIH is feasible and safe in well-selected, appropriately counseled elderly patients.

## 6 Conclusion

Well-selected elderly patients may be candidates for SDD following MIH. Chronologic age alone should not serve as an absolute contraindication to SDD. Elderly patients who underwent SDD were not at increased risk of early post-operative complications or unscheduled presentation within 30 days of discharge after MIH when compared to those admitted for OBS. We did note that elderly patients who also meet objective criteria for frailty may be more vulnerable in the perioperative period, and thus the combination of these factors should be considered by the physician when determining appropriate *peri*-operative monitoring and follow-up of this patient population.

## CRediT authorship contribution statement

**Paulina J Haight:** Conceptualization, Methodology, Software, Validation, Investigation, Data curation, Writing – original draft, Visualization, Project administration. **Rachael N Piver:** Conceptualization, Investigation, Data curation, Writing – original draft. **David A Barrington:** Conceptualization, Methodology, Investigation, Data curation, Writing – original draft, Visualization. **Jae Baek:** Investigation. **Stephen M Graves:** Investigation, Writing – original draft. **Melissa Ardizzone:** Investigation. **Jenifer A Akinduro:** Investigation. **Audrey C Busho:** Investigation. **Deborah Fadoju:** Investigation. **Radhika Pandit:** Investigation. **Raeshawn Stephens:** Investigation. **Lauren M Strowder:** Investigation. **Shreekari Tadepalli:** Investigation. **Brianna VanNoy:** Investigation. **Bhargavi Sriram:** Writing – review & editing. **Eric M McLaughlin:** Formal analysis, Writing – review & editing. **Michelle DS Lightfoot:** Investigation, Writing – review & editing. **Laura M Chambers:** Writing – review & editing. **Kristin L Bixel:** Resources, Writing – review & editing. **David E Cohn:** Resources, Writing – review & editing. **Casey M Cosgrove:** Resources, Writing – review & editing. **David O'Malley:** Resources, Writing – review & editing. **Ritu Salani:** Resources, Writing – review & editing. **Floor J Backes:** Conceptualization, Methodology, Resources, Writing – review & editing, Supervision. **Christa I Nagel:** Conceptualization, Methodology, Resources, Writing – review & editing, Supervision.

## Declaration of Competing Interest

The authors declare that they have no known competing financial interests or personal relationships that could have appeared to influence the work reported in this paper.
